# Screening of Random Peptide Library of Hemagglutinin from Pandemic 2009 A(H1N1) Influenza Virus Reveals Unexpected Antigenically Important Regions

**DOI:** 10.1371/journal.pone.0018016

**Published:** 2011-03-18

**Authors:** Wanghui Xu, Lu Han, Zhanglin Lin

**Affiliations:** Department of Chemical Engineering, Tsinghua University, Beijing, China; Agency for Science, Technology and Research - Singapore Immunology Network, Singapore

## Abstract

The antigenic structure of the membrane protein hemagglutinin (HA) from the 2009 A(H1N1) influenza virus was dissected with a high-throughput screening method using complex antisera. The approach involves generating yeast cell libraries displaying a pool of random peptides of controllable lengths on the cell surface, followed by one round of fluorescence-activated cell sorting (FACS) against antisera from mouse, goat and human, respectively. The amino acid residue frequency appearing in the antigenic peptides at both the primary sequence and structural level was determined and used to identify “hot spots” or antigenically important regions. Unexpectedly, different antigenic structures were seen for different antisera. Moreover, five antigenic regions were identified, of which all but one are located in the conserved HA stem region that is responsible for membrane fusion. Our findings are corroborated by several recent studies on cross-neutralizing H1 subtype antibodies that recognize the HA stem region. The antigenic peptides identified may provide clues for creating peptide vaccines with better accessibility to memory B cells and better induction of cross-neutralizing antibodies than the whole HA protein. The scheme used in this study enables a direct mapping of the antigenic regions of viral proteins recognized by antisera, and may be useful for dissecting the antigenic structures of other viral proteins.

## Introduction

The 2009 A(H1N1) influenza virus, which is also referred to as the swine-origin influenza virus (S-OIV), caused the first global influenza pandemic in recent decades [1]. Given continuous antigenic drift and reassortment of heterotypic influenza viruses circulating in human and animal reservoirs, global concerns have been raised regarding an increasing threat of an influenza pandemic [2]. Current treatment strategies for influenza-A viruses, such as vaccines and drugs, have not provided broad and lasting protection, partly due to the constantly evolving nature of the viral surface glycoprotein, hemagglutinin (HA) that allows it to avoid host immune attack.

HA is the key viral antigen in determining host specificity and inducing neutralizing antibody since it plays a major role in binding to host cell receptors and fusing with host cell membranes [3]. For many challenging diseases caused by viruses, the recognition of certain neutralizing epitopes by the immune system can indeed provide broad and potent protection [4], [5]. The antigenic structure of HA and the corresponding antibody response are not fully understood, complicating rational design of vaccines aimed at modulating antibody responses for targeting key epitopes.

Our previous knowledge of viral antigenic structure was based mainly on the structure of antibody-antigen complexes or mutational analysis of related antigenic drifts [6]–[8]. Recently, many new approaches have emerged for the rapid extraction of monoclonal antibodies toward target antigens from antibody phage display libraries [5], [9], [10], using direct sorting of memory B cells [11], or by immortalization of IgG expressing B cells that reflect the antibody repertoire [12], [13]. These methods provide much more information on viral epitopes, although they are often laborious. The major alternative approaches for epitope mapping are derived from scanning with antigenic peptides displayed on the surface of bacteriophage, bacteria and yeast [14]–[21], which in recent years have increasingly incorporated fluorescence-activated cell sorting (FACS). In these efforts, short defined or random peptides with a narrow length range (<50 amino acid residues (aa)) are used to screen against monoclonal or polyclonal antibodies.

Inspired by advances in cell surface display technology [22], [23] and peptide fragment library construction [24], we devised a high-throughput scheme that utilizes yeast display and FACS that allows for direct screening of random viral peptide libraries against complex antisera instead of isolated antibodies (Zuo T, Shi XL, Xu WH, Lin Z, and Zhang LQ, unpublished results). The peptide library is generated by random digestion of the gene encoding the target viral protein, followed by a PCR-based reassembly step that results in fragments with controllable lengths (normally in the range of 100–500 bp) [24]. These peptides are then expressed on the yeast cell surface by fusion to the yeast adhesion receptor *AGA* 2 protein, which has previously been used to display defined short antigen fragments of various viral and non-viral proteins [19]–[21]. From the sequences of the antigenic peptides after screening, it is possible to determine the relative frequency of each amino acid residue involved in recognition by antisera, and how these residues are distributed in the three-dimensional structure of HA. Using this approach, we analyzed the 2009 A(H1N1) influenza virus HA protein using mouse, goat and human antisera. Unexpectedly, different antigenic profiles were seen for different antisera. Moreover, five antigenic regions were identified, four of which are located in the conserved HA stem region responsible for membrane fusion. ELISA binding assays and absorption experiments using peptides that encompass these regions have confirmed their antigenic activities.

## Results

### Outline of the screening procedure

**Figure 1:**
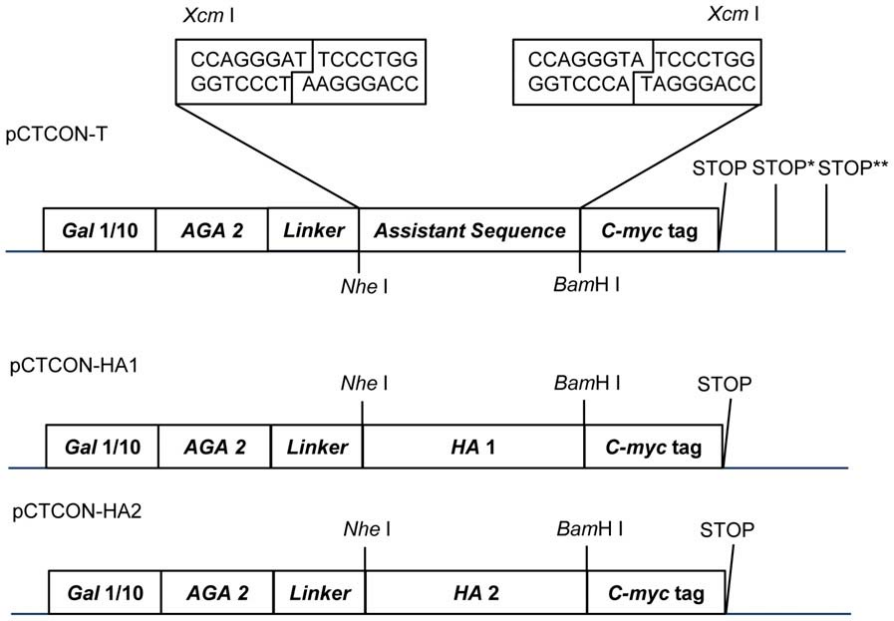
Vectors constructed in the study. Vector pCTCON-T was derived from pCTCON-2 [23] by inserting an assistant sequence with two *Xcm* I sites at both ends between *Nhe* I and *Bam*H I sites. Digestion of pCTCON-T yielded a T vector for TA ligation with random fragments of the H1N1 HA gene. ‘Stop’, ‘Stop*’ and ‘Stop**’ stand for three different stop codons for three possible ORFs, respectively. The random peptide library was expressed under the control of the galactose inducible *GAL* 1/10 promoter and displayed on the surface of the yeast cells by fusion to the yeast adhesion receptor *AGA* 2 protein. Vectors pCTCON-HA1 and pCTCON-HA2, created by inserting HA1 and HA2 genes between the *Nhe* I, *Bam*H I sites of pCTCON-2, were used as positive controls in FCM detection.

The procedure for construction, expression and screening of random viral peptide libraries using yeast surface display is shown in Fig. S1 (see also Materials and Methods for details). In step 1, the full length HA gene of the pandemic 2009 A(H1N1) influenza virus (including the core HA protein of 519 aa, the signal peptide of 16 aa, and the inter membrane domain of 36 aa) was digested and re-assembled by PCR to form a pool of fragments enriched in 100–500 bp [24]. In step 2, the fragment library was ligated into the yeast display vector pCTCON-T (derived from the yeast surface display vector pCTCON-2 [23] shown in Fig. 1), transformed into yeast EBY100 cells and induced for expression, generating a typical library of 1×10^5^–10^6^ clones to cover most of the possible fragments [25]. Because there are 3 stop codons on pCTCON-T downstream of the peptide inserts to terminate every ORF, the theoretical possibility for in-frame fusions is 1/3 for the N-terminal end of the peptide inserts. Thus, the theoretical yield for cells expressing in-frame peptides is 1/6 (the fragments can be inserted in forward and reverse directions).

**Figure 2:**
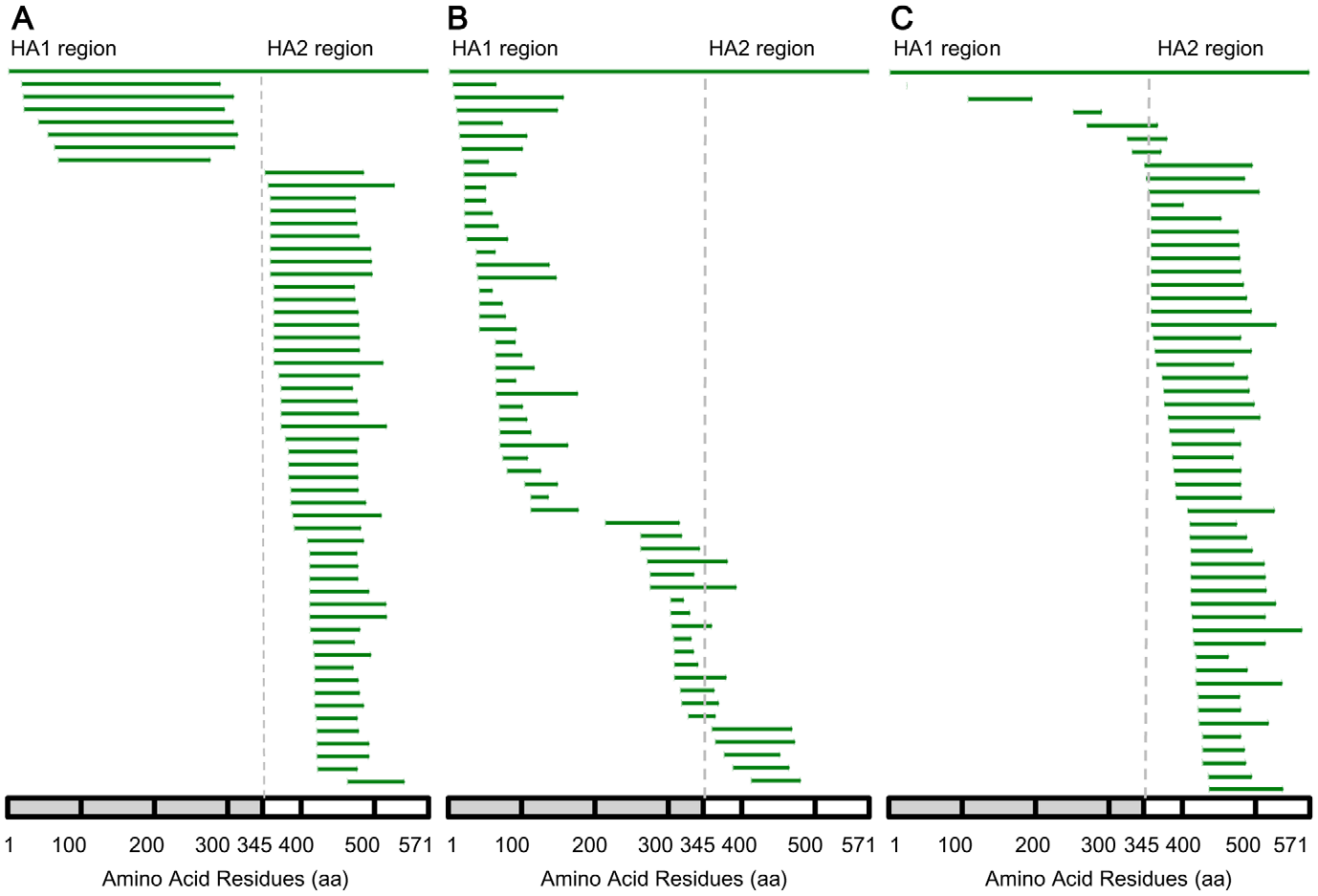
Alignment of antigenic peptide profiles derived from screening against three antiserum samples. (A) 56 peptides (with a RAYS ratio ≥2) obtained using mouse antisera (M-1 to M-56 from top to bottom). (B) 55 peptides obtained using goat antisera with the whole HA protein (G-1 to G-55 from the top to the bottom). (C) 51 peptides obtained using human plasma (H-1 to H-51 from top to bottom). The coordinates of the whole HA protein are indicated by the bars. The borders of the HA1 and HA2 regions (residue 345) are indicated by dashed lines.

**Figure 3:**
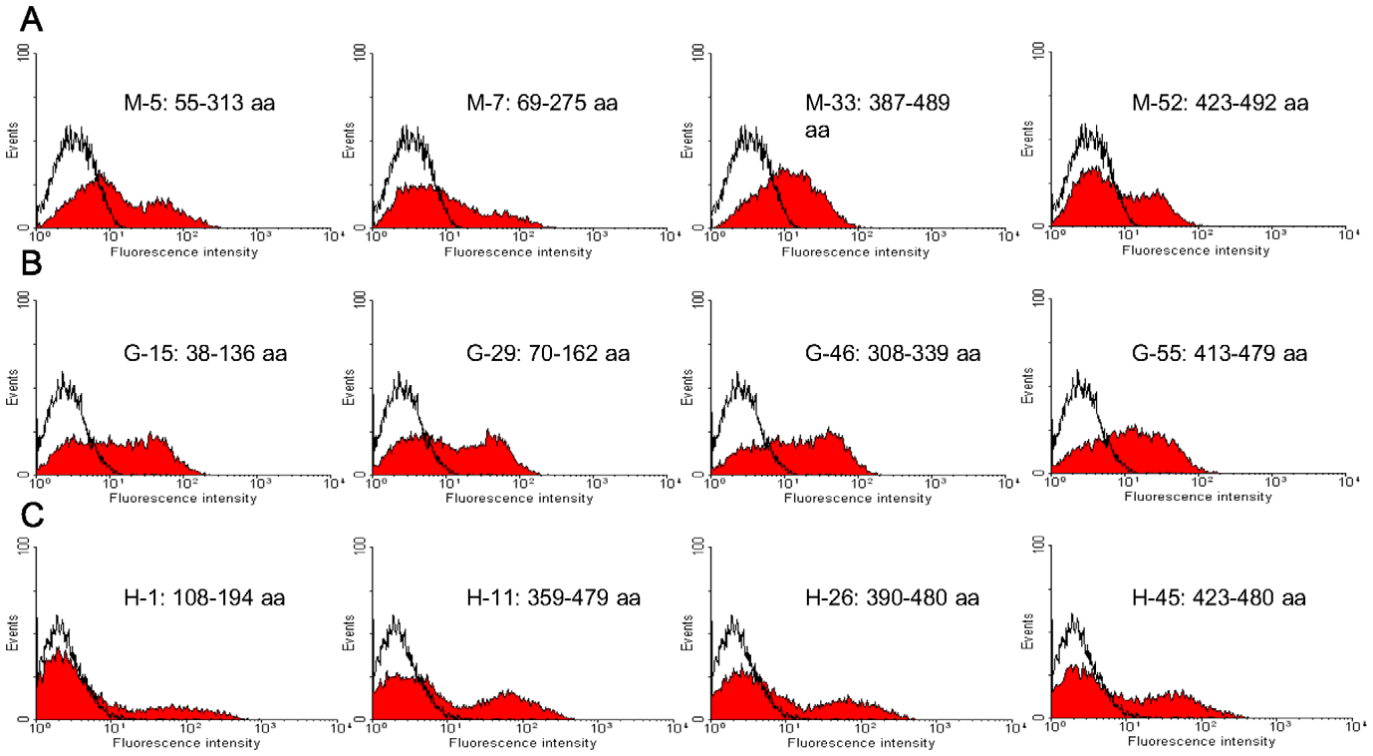
FCM histograms of representative yeast clones. The FCM signal for cells displaying antigenic peptides (the area filled by red color) overlaid with the FCM signal of non-expressing cells containing pCTCON-2 (the black curve) is shown. The designation of the clones follows the same order indicated in Fig. 2. The corresponding HA residues for each clone are illustrated. (A) FCM signals for clones M-5, M-7, M-33, and M-52 from screening against mouse antisera. (B) FCM signals for clones G-15, G-29, G-46 and G-55 from screening against goat antisera. (C) FCM signals for clones H-1, H-11, H-26 and H-45 from screening against human plasma.

**Figure 4:**
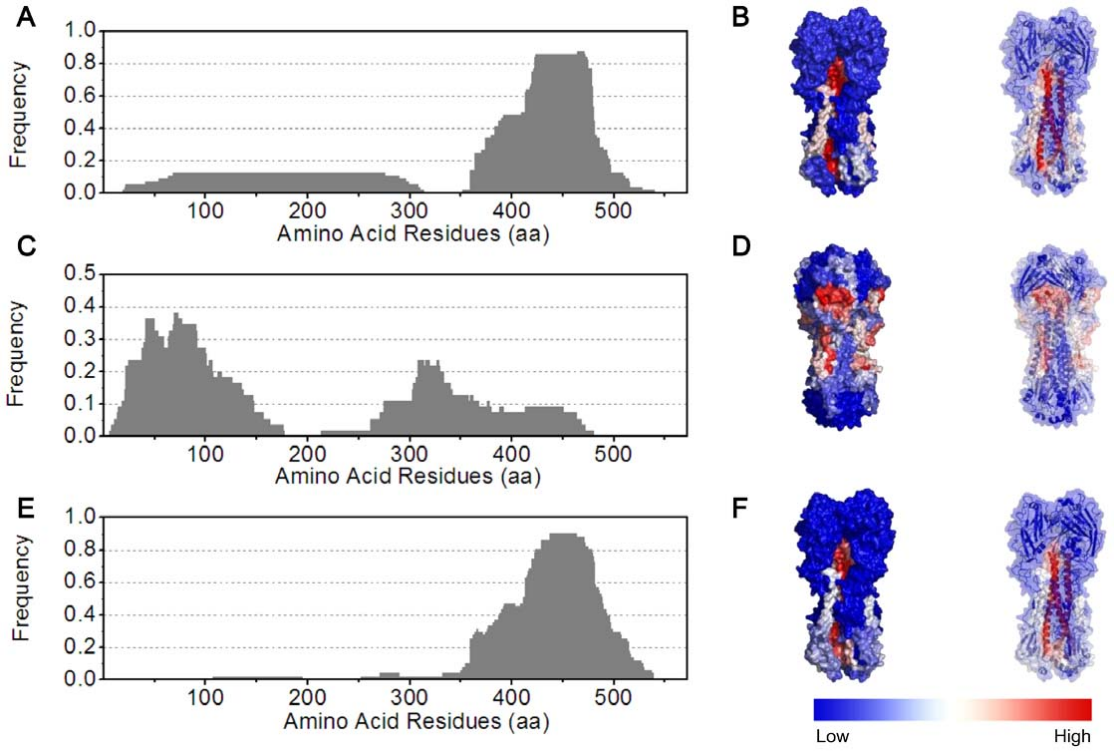
Statistical analyses of antigenic peptides based on screening against three antiserum samples. (A), (C) and (E) illustrate the residue frequency map for the mouse, goat and human antiserum samples, respectively. The x-axis shows amino acid residues of the HA protein. The y-axis shows the normalized frequency of individual amino acid that appears in the pool of antigenic peptides screened against each antiserum sample. (B), (D) and (F) illustrate the structural frequency map for the mouse, goat and human antiserum samples, respectively. Both the surface and backbone structure of the HA trimer of 2009 A(H1N1) virus (PDB ID: 3LZG) are annotated by colors from red to blue, representing a frequency spectrum from high to low, as shown in the color bar.

Subsequently in step 3, the cell library was incubated with antisera, labeled with secondary fluorescent antibody and subjected to FACS in step 4, after which the cells harboring antibody-binding peptides were distinguished from those with non-binding peptides. In step 5, we isolated plasmids directly from the pool of yeast cells containing positive peptide sequences, which were retransformed into *E. coli* for sequencing. This reduced laborious plasmid extraction from individual yeast clones. Plasmids containing in-frame sequences were transformed back into yeast for individual flow cytometry (FCM) verification. Retransformation and FCM were also performed for plasmids containing out of frame sequences (false positive). Antigenic peptide sequences were aligned with the HA sequence in step 6 (see also Figs. 2 and 3). In step 7, statistical analyses both on the sequence and structural levels were then performed to map out the antigenically important regions of the HA protein, by summarizing the frequency of each amino acid residue appearing in the antigenic peptides (see also Fig. 4).

The HA polypeptide is cleaved into two subunits linked by a disulfide bond, HA1 and HA2, to form the mature HA trimer. The majority of the HA1 subunit forms the viral membrane-distal globular head responsible for receptor binding, whereas the HA2 subunit together with the N- and C- termini of HA1 forms the membrane-proximal stem region that plays a major role in membrane fusion. Thus two plasmids, pCTCON-HA1, pCTCON-HA2, respectively, were constructed to display the HA1 subunit of 328 aa (residues 17–344 of the HA protein) and the HA2 ecto-domain of 192 aa (residues 345–536 of the HA protein) as positive controls for FCM (Fig. 1). The HA1 and HA2 displayed on the yeast surface were recognized by the mouse, goat and human antisera, and confirmed by FCM (Fig. S2, panels B, E, and, H; and C, F, and I, respectively).

### Screening of antigenic peptides recognized by mouse antisera immunized with HA protein

We first screened the yeast library displaying the H1N1 HA peptides to study the murine immune response to recombinant HA protein of the 2009 A(H1N1) Influenza virus. Freshly induced library cells were incubated with mixed sera from immunized mice and subsequently labeled with fluorescent secondary antibodies. To avoid potential bias introduced by growth advantage of the yeast cells, only one round of sorting was performed. Plasmids isolated from positive yeast cells were transformed into *E. coli* cells and plated. Single clones (100) were picked randomly and sequenced, of which 82 (82%) were shown to contain in-frame inserts. These results indicate that the sorting process is able to distinguish in-frame sequences from the random library that contained only 1/6 (16.7%) in-frame inserts efficiently. After verification of individual peptides by FCM detection, the ratio of recombinant antigen expressed on the yeast surface (RAYS) was determined by dividing the mean fluorescence intensity of peptide expressing yeast cells by the mean fluorescent intensity of the non-expressing (pCTCON-2) yeast cells [26]. 56 positive clones with a RAYS ratio ≥2 were considered as antigenic and analyzed at both the sequence and structural level [26]. Alignment of positive peptides with the original HA sequence shows that 87% are in the HA2 region (95±27 aa), with just 13% in the HA1 region (259±26 aa) which cover nearly the entire HA1 protein (Fig. 2, panel A). The FCM histograms of several representative yeast clones are shown in Fig. 3, panel A. Clones M-5 (corresponding to residues 55–313 of HA, or 70 aa shorter than HA1) and M-7 (residues 69–275, or 120 aa shorter than HA1) were strongly positive (RAYS ratio >5), and contained the entire receptor binding domain (RBD) and the vestigial esterase domain in the globular HA head. Clones M-33 (residues 387–489) and M-52 (residues 423–492) displayed much shorter peptides but also showed strong fluorescent FCM signals (Fig. 3, panel A), albeit with relatively lower RAYS ratios of 3–4. Judged from the mean fluorescence intensities, the binding affinities of these displayed peptides were generally weaker than the whole HA1 (Fig. S2, panel B).

The sequencing results of the antigenic peptides alone are less informative (Fig. 2). Nevertheless, when we perform a statistical analysis by summarizing the normalized frequency of each amino acid residue in all positive antigenic peptides (defined as the residue frequency map), a major peak with the full width at half maximum (FWHM) from residues 387–480 is revealed (Fig. 4, panel A). We also modify the residue frequency map by taking into account the respective RAYS ratios of positive peptides, which however results in an overall rather similar frequency map (Fig. S3, panel B). Furthermore, even if we combine all the in-frame sequences from sorted yeast cells, a similar map (Fig. S3, panel C) is again obtained, implying that the noises from the in-frame but false positive peptides are minimal. This could be useful if one wishes to gain a quick view of the “hot” antigenic regions.

**Figure 5:**
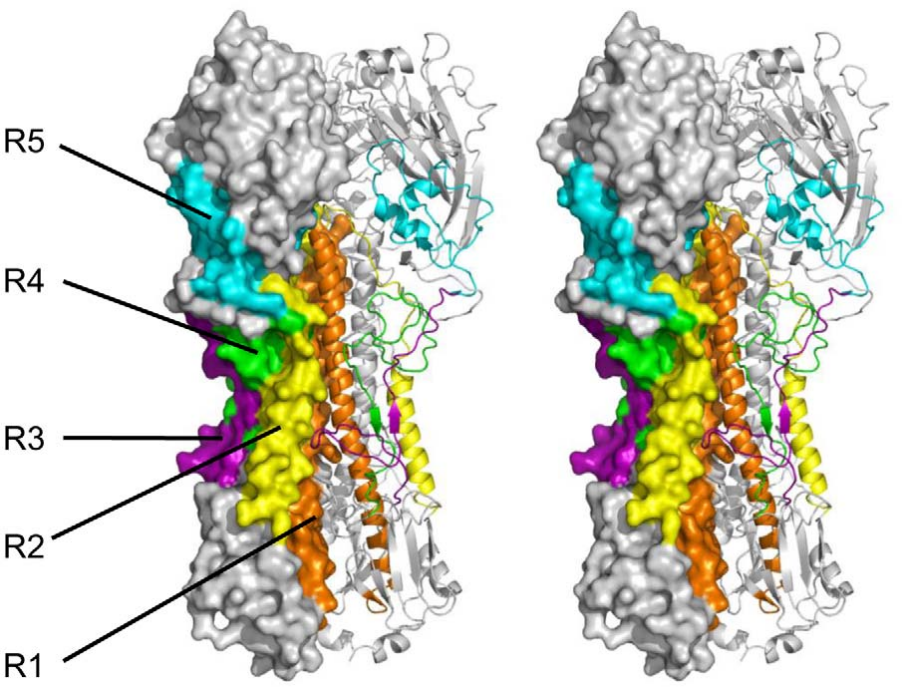
The five dominant antigenic regions displayed in stereo view. The HA monomer surface view is shown on the left and colored to illustrate the five dominant antigenic regions. In order from most membrane proximal to distal: R1 (orange, residues 424–480 of HA) and R2 (yellow, residues 387–423) in HA2 were determined by screening against mouse and human antisera; R3 (purple, residues 22–60), R4 (green, residues 303–350), and R5 (cyan, residues 61–125) in HA1 were determined by screening against goat antisera. The HA monomer cartoon view is shown on the right and follows the same coloring scheme, with the third monomer shown in the back and colored in grey.

The residue frequency map is then overlaid onto the crystal model of the trimeric HA of the 2009 A(H1N1) influenza virus (PDB ID: 3LZG) [27], and annotated by colors from red (high frequency) to blue (low frequency) (Fig. 4, panel B). This structural frequency map reveals that the major peak (corresponding to residues 387–480 within the FWHM) on the residue frequency map can be divided into two dominant antigenic regions since they have independent structural features. One corresponds to the central coiled-coil helix CD in the HA2 subunit responsible for membrane insertion, designated as R1 (residues 424–480, indicated by red, see also Fig. 5), while the other contains helix A in the HA2 subunit that is important for conformational change upon low pH exposure, designated as R2 (residues 387–423, indicated by faint red and white, see also Fig. 5) [28]. R1 is present in a relatively recessed surface area while R2 is located near the exterior surface area of the stem region. For the HA1 subunit, no distinct antigenic region is identified, because of the limited number of peptides (7) with relatively longer lengths (all >200 aa), which are insufficient to form peak areas.

### Screening of antigenic peptides recognized by goat antisera immunized with pandemic 2009 A(H1N1) influenza virus

We also screened the same yeast library against serum samples from goats immunized by the 2009 A(H1N1) influenza virus to examine whether different animal models would yield a similar profile of antigenic peptides. Following sorting and sequencing, 78/100 clones were confirmed as in-frame, among which 55 tested positive by the goat anitsera in FCM (RAYS ratio ≥2). Surprisingly, alignment of the positive antigenic peptides with the full HA protein shows a distribution strikingly different from the alignment obtained using the mouse antisera in several aspects. First, 78% of the antigenic peptides are located in the HA1 region and have shorter lengths (54±32 aa), while only 9% are in the HA2 region (96±19 aa). Second, 13% of the peptides are in the region across HA1 and HA2 (66±31 aa) (Fig. 2, panel B). Representative antigenic clones were subjected to FCM. As shown in Fig. 3, panel B, clones G-15 (residues 38–136 of HA) and G-29 (residues 70–162) in the HA1 region, G-46 (residues 308–339) near the HA1-HA2 cross region, and G-55 (residues 413–479) in the HA2 region, all shorter than 100 aa, demonstrated strong antigenicity (RAYS ratio >5). Judged from the mean fluorescence intensities, the binding affinities of these displayed peptides were generally stronger than the whole HA1 (Fig. S2, panel E).

By analyzing the residue frequency map, more peaks are revealed than those obtained from the mouse anitsera, with the largest one with its FWHM spanning residues 22–125 with a small split around residue 60, and a medium one with its FWHM at residues 303–350 (Fig. 4, panel C, and Fig. S4). In the structural frequency map (Fig. 4, panel D), the largest peak is composed of two independent regions: residues 22–60 at the N-terminus of HA1 adjacent to helix A on HA2, designated as R3; and residues 61–125 located in the vestigial esterase domain of the globular head, designated as R5 (indicated by two red regions in the globular head and stem region, respectively, see also Fig. 5) [6]. The antigenic region of the medium peak (residues 303–350) consists of the C- terminal extension of HA1, designated as R4 (indicated by faint red within the stem region, see also Fig. 5). It should be noticed that R1 and R2 identified in the mouse serum screening also appear in this residue frequency map, although in a much less dominant manner as illustrated by the small plateau between the end of R4 and residue 479 (Fig. 4, panel C). Taken together, the antigenic peptides recognized by the goat antisera are different from those recognized by the mouse antisera, and in particular present a more diverse picture of antigenic sites in the HA1 region.

### Screening of antigenic peptides recognized by human plasma immunized with the 2009 A(H1N1) influenza vaccine

The screening against human plasma immunized by the 2009 A(H1N1) influenza vaccine was carried out essentially as described above. Among the 100 sequences obtained after FACS analyses, 74 peptides were found to be in-frame, and 51 were confirmed to be antigenic (RAYS ratio ≥2). It is interesting to see that the distribution of theses peptides is similar to that obtained using the mouse antisera, with only 4% of peptides in the HA1 region (87 and 37 aa in length), 4% across HA1 and HA2 (96 and 39 aa in length), and 92% in the HA2 region (100±30 aa) (Fig. 2, panel C). Using FCM, three representative clones, H-11 (residues 359–479 of HA), H-26 (residues 390–480) and H-45 (residues 423–480), all in the HA2 region, showed strong signals (RAYS ratio >5). Although only 2 antigenic peptides are in the HA1 region, a clone expressing H-1 (residues 108–194), which harbored the Sa antigenic site in RBD of the HA protein, yielded a RAYS ratio greater than 3 (Fig. 3, panel C). Again, judged from the mean fluorescence intensities, the binding affinities of these displayed peptides were generally weaker than the whole HA1 (Fig. S2, panel H).

The residue frequency map also reveals a major peak (FWHM at residues 392 to 480). This peak position is similar to that obtained using the mouse antisera (FWHM at residues 387–480), resulting in an almost identical structural frequency map (Fig. 4, panels E and F, and Fig. S5). Thus, these antigenic regions are considered identical to R1 and R2 obtained from the mouse serum screening (Fig. 5).

### Accessibility of antigenic peptides displayed on yeast cell surface

The accessibility of short antigenic peptides displayed on the yeast cell surface [19]–[21] was confirmed in this study with fluorescence confocal microscopy, as exemplified by the antigenic clones G-29 (93 aa) and G-46 (32 aa), from the screening against the goat antisera (Fig. S6). As shown in the figure, the antigenic peptides were localized on the outer surface of the membrane, and well accessible to the antibodies in the complex antisera.

### Neutralization assay using yeast cells displaying antigenic peptides

Three representative yeast clones displaying antigenic peptides G-15, G-55, and H-11, obtained from the screening against the goat antisera and human plasma samples, were characterized for neutralization titers (IC_50_) by testing protection efficacy to MDCK cells from the A/reassortant/NYMC X-179A/California/07/2009(H1N1) exposure. Cells containing pCTCON2 were used as absorbent controls for evaluation of background IC_50_ values. HA1 and HA2 were also incorporated to determine the ability of the globular head and stem regions to absorb antibodies in the antisera. As summarized in Table S1, all single clones reduced the IC_50_ of the corresponding antisera, demonstrating qualitatively the neutralization efficacy of these antigenic peptides.

### Binding affinities of purified peptides to mouse, goat and human antisera

**Figure 6:**
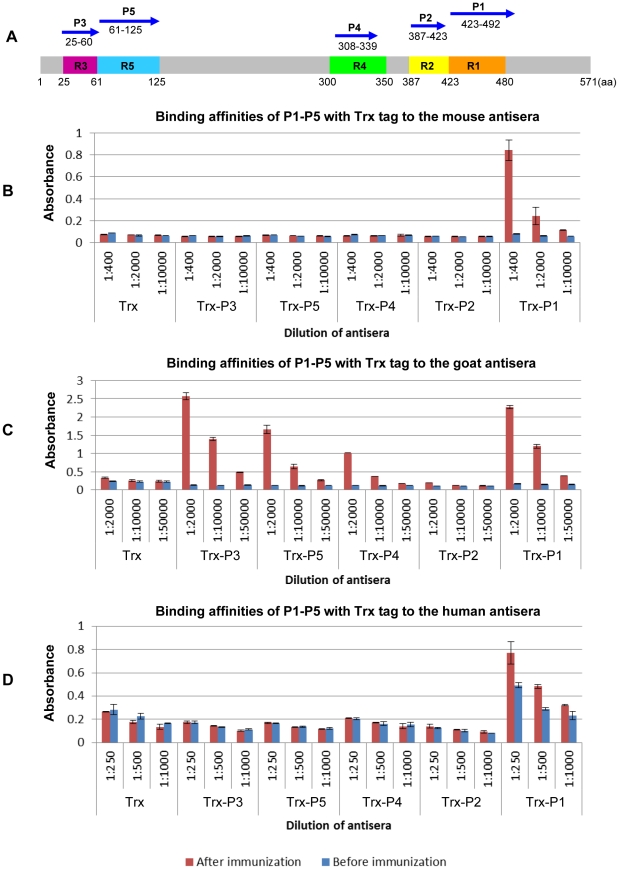
Binding affinities of five representative peptides to three antisera. (A) Amino acid positions of the newly designed peptides P1–P5 (blue arrows), in relation to the five antigenic regions R1–R5. The coordinate of the HA protein is indicated by the grey bar, with the five antigenic regions represented in the same color as in Fig. 5. The peptides P1–P5 were expressed as C- terminal fusions to the thioredoxin (Trx) tag. Binding activities of the peptides against the mouse (B), goat (C) and human (D) antisera before and after immunization were characterized by the method of ELISA, with the Trx protein as the negative control. The x-axis shows the dilution ratios of corresponding antiserum samples. The y-axis shows the absorbance at 450 nm after development with the substrate 3,3′,5,5′-tetramethylbenzidine (TMB).

To further confirm the antigenic activities of the identified R1–R5 regions, five newly designed short peptides encompassing regions R1–R5 (Fig. 6, panel A) were expressed as C- terminal fusions to thioredoxin (Trx) and purified from *E. coli* [29], [30]. The binding affinities of these peptides to the mouse, goat and human antisera were characterized by the method of ELISA, with thioredoxin as the control (Fig. 6, panels B and D). As can been seen from the figure, the ELISA results generally correspond well to the screening results shown in Figs. 4 and 5. Specifically, the peptide P1 (corresponding to the R1 region identified in all three screenings, Figs. 4 and 5) was reactive to all three antisera. The peptides P3, P4 and P5, corresponding to the R3–R5 regions identified only in the screening against the goat antisera (Fig. 4, panels C, D, and Fig. 5) were only reactive to the goat antisera (Fig. 6, panel C). There is one exception in that the peptide P2 (corresponding to the R2 region identified in the screenings using the mouse and human antisera, Figs. 4 and 5) showed no measurable affinity to either mouse or human antisera, suggesting that the R2 region might be less conformation-independent than the other four regions.

It should be noted that, after subtraction of the background binding of the negative control protein (Trx), the binding affinity of the peptide P1 to the human antisera after vaccination increased only about one fold compared with the antisera before vaccination (Fig. 6, panel D). This might be contributed to cross-reactive antibodies to other influenza subtypes pre-existing in the human serum subjects, given the conservative nature of the P1 sequence or the R1 region [13].

## Discussion

We report here the identification of antigenic peptides of the 2009 A(H1N1) influenza HA protein from a combinational library of viral protein fragments displayed on the surface of yeast cells and sorted by FACS. The peptide fragments were constructed in a way to have a broad but limited range of lengths [24], and screened using antisera from mouse, goat and human. We then used a novel statistical approach to identify antigenically important regions of the viral protein based on the frequency of each residue appearing in the antigenic peptides at both the primary sequence and structural level.

This approach reveals interesting antigenic features of the HA protein. First, the antibody responses to the 2009 A(H1N1) viral protein HA appear to vary significantly depending on the species, with the goat response being strikingly different from those of mouse and human (Fig. 4). These results imply possibly different immune responses among animal species, and in the case of human, the specific immunization background may also play a role [13]. Moreover, the antigenic peptides obtained from the screening against the mouse or human antisera are predominantly located in the HA2 region, whereas those from the screening against the goat antisera are predominantly in the HA1 region. Second, five antigenically important regions, R1 to R5 in the order from most membrane proximal to distal (Fig. 5), are identified. Moreover, except for R5 which is located in the vestigial esterase domain of the HA globular head, all of the other regions are in the HA stem region. Among them, R1 and R2 come together to form a major peak with a FWHM range from residues 387 to 480 (Fig. 4, panels A and E). Although the peptide P2 corresponding to the R2 region showed no measurable affinity to either mouse or human antisera (Fig. 6), which suggests that this region might be more conformation-dependent than the rest four regions, it is assigned as a separate region for the following reasons: (1) structurally it is distinguishable from the R1 region (Fig. 5), and (2) several recent studies have reported the epitopes of monoclonal antibodies targeting H1 subtype HA proteins, which fall into the R2 region of this study [10], [31], [32] (see below).

Previously most influenza HA antibodies have been found to recognize epitopes in the globular head and interfere with binding to target cells [6]–[8], [33]–[36], for example, the classical five antigenic sites (Sa, Sb, Ca_1_, Ca_2_, and Cb) [6], [7]. However, technically the monoclonal antibodies used in these previous studies were obtained from murine hybridoma cells by radioimmunoassay (RIA) or hemagglutination inhibition (HI) assay. The nature of these assays favored the isolation of antibodies that bound predominantly to the globular head of the virus and inhibited binding of the virus to the host cells. In this current study, however, complex antisera were used, which should contain antibodies recognizing other regions of the HA protein. On the other hand, only one antigenic region (R5) is identified in the globular head in this study, possibly because the epitopes in this head region are more conformation-dependent and may require longer peptides or even a complete domain of HA1 to be displayed for full antigenicity (at least for the mouse serum and human plasma samples). It is then noteworthy that the head region, especially the area around the receptor binding site, contains mostly beta structures, which more likely require stabilization by long-range interactions than the helical structures in other regions of HA (Fig. 5). However, the peptide library constructed in this study was enriched at 100–500 bp (or less than 170 aa), which was likely insufficient to map the conformational epitopes. Nonetheless, the current screening method is a valuable complementation to the more classical approaches that are biased toward full HA1 or HA2 domain.

Several recent studies support our findings regarding antigenic regions R1–R4 in the stem region of HA. In efforts aimed at characterizing antibodies with cross-neutralizing activity for various influenza virus subtypes [10], [13], [31], it was found that most of these antibodies bind to the HA stem region and interfere with conformational changes of the protein critical for membrane fusion. For example, in the case of antibody CR6261 (binding to HA of the SC1919 virus, H1 type), two antigenic regions on HA stem were revealed: helix A in the HA2 region, and the adjacent HA1 region [32]. The critical sites in helix A (corresponding to residues 390–391, 393–395, 398, 401–402, 405 in H1N1 HA) fall within the R2 region identified in this study, whereas the hydrophobic interaction sites in the adjacent HA1 region (corresponding to residues 38–42, 306–307 in H1N1 HA) fall into regions R3 and R4 of this study, respectively. More recently, it was reported that for a different cross-neutralizing antibody that targets a broad range of H3 subtype virus strains, 12D1, the dominant contacts on the antigen (corresponding to residues 425–455 in H1N1 HA) [37] correlate well with the R1 region found in this study. Furthermore, vaccination in mice using a HA2-based synthetic peptide mimicking the 12D1 epitope was shown to provide protection against influenza viruses of H3N2, H5N1, and H1N1 subtypes [38]. Taken together, our approach should be useful for dissecting antigenic regions on the HA stem, and providing clues for designing more potent peptide vaccines in inducing cross-neutralizing antibodies than the entire HA protein [32].

Compared with other labor-intensive epitope identification methods that rely on identifying critical residues in escape mutants and crystal structures, the method used here gives a direct and panoramic mapping of the antigenic regions of viral proteins recognized by antisera in a facile and high-throughput way. Our approach likely contains an inherent bias for shorter peptides, but nonetheless complements the more classical approaches that favor longer antigenic peptides or full antigenic domains. The scanning of antigenic peptides based on phage, bacterial or yeast surface display methods has been increasingly used in recent years. Our approach presents two advantages. First, technically we adopt an easy-to-implement scheme for generating a controllable size of peptides and our screening typically involves only one round of FACS-based sorting, in contrast to multiple rounds of panning or sorting used in the literature [15], [16], [18]. Although the latter results in many repeat sequences, we argue that much information is lost in the enrichment process. Second and more importantly, since simple alignment of antigenic peptides with the corresponding viral protein yields only limited information [15], [17], [39], we employ a novel statistical means of summarizing the frequency for each residue appearing in the antigenic peptides at both the primary sequence and structural level, which enables a direct mapping of the antigenic structure of a viral protein. Along this line, it is interesting to note that, using our statistical approach, we re-evaluate the highly repeated antigenic peptides identified for the H5N1 HA protein in a recent report [15]. Several major peaks are clearly revealed (Fig. S7), which should be useful for further characterization of the antigenic peptides for H5N1 HA.

## Materials and Methods

### Materials

Mouse antisera, kindly donated by Dr. Zhonghua Liu, AIDS Research Center, School of Medicine, Tsinghua University (Beijing, China), were derived by immunizing mice with 293T-cell secreted recombinant HA protein from A/California/04/2009(H1N1) virus. Hyperimmune goat sera, a gift from Dr. Guoyang Liao, Institute of Medical Biology of Chinese Academy of Medical Sciences and Peking Union Medical College (Kunming, China), were raised against a Chinese pandemic strain of 2009 A(H1N1) influenza virus. Human plasma samples were obtained 3 weeks post vaccination with inactivated 2009 A(H1N1) influenza vaccine, and a mixture of 9 samples with the highest activity among 110 samples as judged by ELISA. These samples were provided by Dr. Boping Zhou of Shenzhen East Lake Hospital (Shenzhen, China), and written informed consent was obtained from all study participants. All samples were de-identified prior to analysis, and the protocols were approved by the Institutional Review Board (IRB) of Shenzhen East Lake Hospital. All viral strains are closely related.

Restriction enzymes and DNA polymerases were purchased from New England Biolabs (Beverly, MA) or Takara (Dalian, China). DNase I was obtained from Worthington (Lakewood, NJ). Fluorescence-labeled secondary antibodies were purchased from Invitrogen (Shanghai, China) or Santa Cruz Biotechnology (Santa Cruz, USA). Oligonucleotides were synthesized by Invitrogen or Takara. The kits for DNA purification, gel recovery, plasmid miniprep and A-Tailing modification were obtained from Tiangen (Beijing, China) or Takara (Dalian, China). The kit for yeast plasmid DNA isolation was from Omega Biotech (Victoria BC, Canada). Sequencing was performed by Invitrogen or by SinoGenoMax (Beijing, China). *Escherichia. coli* DH5α was obtained from Takara. Yeast strain *Saccharomyces cerevisiae* EBY100 was obtained from Invitrogen.

### Construction of yeast surface display vectors

The pCTCON-2 yeast display vector was kindly provided by Prof. Dane Wittrup [23]. The DNA fragments coding the HA1 and HA2 proteins were amplified from plasmid CMV-R-Cali-04-09 carrying the whole HA gene of A/California/04/2009(H1N1) virus, provided by Prof. Paul Zhou, Institute Pasteur of Shanghai, Chinese Academy of Sciences (Shanghai, China). DNA fragments were digested and inserted between the *Nhe* I and *Bam*H I sites of pCTCON-2, resulting in the plasmid pCTCON-HA1 and pCTCON-HA2. The primers used for HA1 and HA2 amplification was as follows: HA1-For, 5′-ACAAT***CATATG***GCTGACACACTGTGCATCGGA-3′; HA1-Rev, 5′-CTAAC***AAGCTT***TCTGCTCTGGATGCTAGGGAT-3′; HA2-For, 5′-ACAAT***CATATG***GGACTGTTCGGAGCTATCG-3′; HA2-Rev, 5′-CTAAC***AAGCTT***GATCTGGTAGATTCTTGTG-3′ (*Nhe* I and *Bam*H I sites are in bold and italic).

Two *Xcm* I restriction sites (CCANNNNN/NNNNTGG) were introduced between *Nhe* I and *Bam*H I sites of pCTCON-2 to create pCTCON-T. Digestion of pCTCON-T by *Xcm* I restriction nuclease and gel extraction yielded the T-vector with two 3′ T overhangs. Random fragments with 3′ A tails were inserted between the two *Xcm* I sites by T-A ligation.

### Preparation of HA fragment library

The gene coding for the full HA protein of A/California/04/2009(H1N1) virus was amplified from plasmid CMV-R-Cali-04-09 with the forward primer 5′-CGCCACCATGAAGGCTATCC-3′ and reverse primer 5′-TTAGATGCAGATTCTGCACTG-3′. Detailed procedures for fragmentation and reassembly were described previously, except that Phusion® high-fidelity polymerase (NEB) was used in reassembly [24]. The reassembled DNA samples were purified and modified to add 5′ A overhangs by an A-Tailing Kit (Takara). The backbone vector pCTCON-T was digested with *Xcm* I and purified with a Tiangen gel purification kit to reduce self-ligation. The gene fragments and backbone vector were ligated at 16°C for 16 h and then electroporated into *E. coli* DH5α competent cells for propagation. The plasmid library was then isolated and used to transform yeast EBY100 cells by the Li-Ac method [40]. Expression of peptide libraries on yeast EBY100 cells was performed as reported by Wittrup's group [23]. Transformed yeast cells were grown for 24 h at 30°C with shaking in SDCAA medium (yeast nitrogen-based casamino acid medium containing 20 g⋅L^−1^ glucose) and passed one time into fresh medium to eliminate dead cells. The library cells were then centrifuged, resuspended in SGCAA medium (yeast nitrogen-based casamino acid medium containing 20 g⋅L^−1^ galactose) to an OD_600_ value of 0.5–1 and induced at 20°C for 36–48 h with shaking.

### Fluorescence labeling, FACS-based screening and characterization of antigenic peptides

Prior to yeast library screening, nonspecific interactions of serum samples (i.e., mouse, goat or human antisera) with yeast proteins were removed by incubation with induced yeast cells carrying the control vector pCTCON-2. To label cells for FACS, 1×10^7^ (For yeast EBY1001 cells, OD_600_≈1×10^7^ cells/mL) freshly induced library cells were pelleted at 8,000 g for 1 min in a 1.5 mL microcentrifuge tube and washed two times with 1 mL of 1×Phosphate Buffered Saline (PBS). Cells were then incubated with 100 µL of pre-absorbed serum (1∶50 diluted in PBS) at 4°C for 1 h. Unbound antibodies were removed by washing two times with 1 mL of PBS. The library cells were incubated with 1–2 µg of fluorescein isothiocyanate (FITC) or phycoerythrin (PE) labeled secondary antibodies in 100 µL at 4°C for 45 min. After washing two times with PBS, cells were resuspended in 1 mL of PBS and loaded onto a BD-FACS AriaII™ machine (Shanghai, China) for sorting. Yeast cells harboring the pCTCON-2 vector were processed as described above and used as a negative control. The positive gate was set to exclude all negative control cells (<0.1% leakage). The total amount of cells used for sorting was adjusted to ensure that more than 1×10^5^ cells could be harvested, which were then collected in 5 mL of SDCAA medium and grown overnight at 30°C with shaking.

After one passage of library cells into fresh medium, heterogeneous plasmid DNA was isolated from the library and transformed into *E. coli* DH5α cells. For each library, a total of 100 colonies were randomly picked from the plates and sequenced using a pair of primers (pCTCON2-Seq-For: 5′-GTTCCAGACTACGCTCTGCAGG-3′ and pCTCON2-Seq-Rev: 5′-GTTCCAGACTACGCTCTGCAGG-3′). The plasmids carrying gene fragments inserted in-frame with the original HA gene were re-transformed into yeast EBY100 cells, induced, and labeled as described for FCM detection on a BD-FACS Calibur cytometer (Shanghai, China). The ratio of recombinant antigen expressed on the yeast surface (RAYS) was determined by dividing the mean fluorescence intensity of peptide expressing yeast cells by the mean fluorescence intensity of the non-expressing (pCTCON-2) yeast cells. Clones with a RAYS ratio ≥ 2 were considered positive and the corresponding peptide sequences were selected for further analysis [26].

### Fluorescence Confocal Microscopy

Yeast cells containing plasmids for displaying antigenic peptides were grown at 30°C for 24 h in SDCAA medium and induced at 20°C for 36 h in SGCAA medium. 1×10^7^ cells were harvested by centrifugation, incubated with goat antisera (1∶100 diluted in 100 µL of PBS) and labeled with 1 µg of FITC conjugated anti-goat IgG secondary antibody as described above. Labeled cells were resuspended to 5×10^7^ cells/mL, fixed with 4% paraformaldehyde and photographed on a Zeiss 710 laser scanning confocal microscopy (Carl Zeiss, Germany) at the Center of Biomedical Analysis, Tsinghua University. Yeast cells containing non-expressing vector pCTCON-2 and HA1-expressing vector pCTCON-HA1 were treated in the same way as negative and positive controls, respectively.

### ELISA assays for purified representative peptides against three antisera

Five peptides newly designed to encompass the regions R1–R5 were expressed as fusions to the C- terminus of thioredoxin (Trx) and purified from *E. coli*, using a modification of the self-cleaving elastin-like polypeptide tag method (ELP) (with purity over 80% as judged by SDS-PAGE) [29], [30]. For comparison, the Trx tag was used as the negative control. When mouse and goat antisera were employed as the first antibody, the wells were coated with 100 ng of peptides or Trx. But for human antisera, 10 ng of peptides or Trx control was used to decrease the background signal caused by unspecific interactions between the human antisera and the trace impurities from *E. coli* cells. After blocking with 10% Fetal Bovine Serum (FBS) diluted in 1×PBS with 0.25% Tween-20 (PBST), serial dilutions were added to each well and incubated for 1 h at 37°C, followed by addition of 1∶5,000 diluted HRP-conjugated secondary antibody. Assays were developed by adding 100 µL of 3,3′,5,5′-tetramethylbenzidine (TMB) substrate solution and the reactions were stopped with 50 µL of H_2_SO_4_ (1 M). The assays were carried out in duplicates and each well was washed 4 times with PBST between steps. The absorbance at 450 nm was recorded on a SpectroMAX 190 Microtiter reader (Molecular Devices, CA).

### Neutralization assay

Prior to the Neutralization assay, 500 µL antisera (diluted 1∶50, and pre-absorbed with induced yeast cells carrying the control vector pCTCON-2) were mixed with **f**reshly induced yeast cells (1×10^8^) displaying specific antigenic peptides on surface. After incubation at 4°C for 12 h, the mixtures were centrifuged at 8,000 g for 1 min and supernatants mixed with another batch of freshly induced yeast cells. This process was repeated four times, and then the supernatants were used for determining the neutralization titers, using a microneutralization assay with A/reassortants/NYMC X-179A/California/07/2009(H1N1) virus and MDCK cells according to standard procedures [41]. Briefly, triplicate serial dilutions (1∶50 to 1∶1600) of antiserum samples were incubated with 100 50% tissue culture infective dose (TCID_50_) of virus for 2 h at 35°C prior to adding MDCK cells. Cells were incubated at 35°C for 72 h, and the half maximal inhibitory concentration (IC_50_) was determined. All the neutralization assays were performed at the AIDS Research Center, School of Medicine, Tsinghua University following standard procedures.

## Supporting Information

Figure S1 **Schematic outline of the screening approach.** In Step 1, the gene encoding the viral protein H1N1 HA was amplified, digested and re-assembled to generate the fragment library (the red and grey segments indicate the antigenic and non-antigenic peptides, respectively). In Step 2, the fragment library was ligated into the display vector pCTCON-T, transformed into yeast cells and induced for expression. In Step 3, the yeast cells expressing random peptides were incubated with antisera and fluorescence-labeled second antibodies and subjected to FACS in Step 4. Afterward in Step 5, the gene fragments were isolated from the sorted cells and sequenced. The in-frame sequences were re-transformed into yeast cells and verified by FCM detection individually. In Step 6, an antigenic peptide profile was extracted for the sequences corresponding to the antigenic peptides, based on which the residue frequency and structural frequency maps were derived in Step 7.
(TIF)

Figure S2 **FCM detection of HA1 and HA2 displayed on yeast.** Panels A, D, and G show FCM histograms of yeast cells expressing negative control vector pCTCON-2 stained with mouse antisera (and labeled by anti-Mouse IgG FITC), goat antisera (labeled by anti-Goat IgG FITC), and human plasma samples (labeled by anti-Human IgG PE), respectively. HA1 positive controls are similarly shown in panels B, E and H, and HA2 positive controls in panels C, F and I.
(TIF)

Figure S3 **Statistical analyses of peptides from screening against mouse antisera.** (A) Frequency map for each residue appearing in the 56 positive antigenic peptides (RAYS ratio ≥2). This figure is same as Fig. 4, panel A. (B) Frequency map for each residue appearing in the 56 positive antigenic peptides, but weighted by the respective RAYS ratio. (C) Frequency map for each residue appearing in all 82 in-frame peptides sorted from the library before they were individually verified by FCM.
(TIF)

Figure S4 **Statistical analyses of peptides from screening against goat antisera.** (A) Frequency map for each residue appearing in the 55 positive antigenic peptides (RAYS ratio ≥2). The figure is same as Fig. 4, panel C. (B) Frequency map for each residue appearing in the 55 positive antigenic peptides, but weighted by the respective RAYS ratio. (C) Frequency map for each residue appearing in all 78 in-frame peptides sorted from the library before they were individually verified by FCM.
(TIF)

Figure S5 **Statistical analyses of peptides from screening against human plasma.** (A) Frequency map for each residue appearing in the 51 positive antigenic peptides (RAYS ratio ≥2). The figure is same as Fig. 4, panel E. (B) Frequency map for each residue appearing in the 51 positive antigenic peptides, but weighted by the respective RAYS ratio. (C) Frequency map for each residue appearing in all 74 in-frame peptides sorted from the library before they were individually verified by FCM.
(TIF)

Figure S6 **Fluorescence confocal microscopic images of yeast cells displaying antigenic peptides.** Binding of the antibodies in the goat antisera to the yeast cells displaying the control vector pCTCON-2 (A), HA1 (B), and antigenic peptides G-29 (C), G-46 (D) (see also Fig. 3, panel B) were visualized by using a FITC-labeled anti-goat IgG secondary antibody. The lengths of the antigenic peptides are shown in brackets.
(TIF)

Figure S7 **Statistical analyses of antigenic peptides based on the phage panning results shown by Khurana et al.** [**15**] **.** The x-axis represents all H5N1 HA amino acid residues. The y-axis shows the normalized frequency of individual residue appearing in the 784 antigenic peptides (39 unique sequences) obtained from panning against H5N1 avian influenza convalescent sera. The six clusters (I–VI) defined by Khurana et al. are graphically represented below the x-axis. Several representative antigenic peptides are also shown as green arrows (numbered according to Khurana et al.). Even though the antigenic peptides were enriched multiple times during the screening process and thus these peptides are less diverse and might be biased in sequences, several peaks are clearly identifiable, and predominantly in the HA2 region.
(TIF)

Table S1 H1N1 virus neutralization assay.
(DOC)
