# Supplementary figures and images for: Screening of Random Peptide Library of Hemagglutinin from Pandemic 2009 A(H1N1) Influenza Virus Reveals Unexpected Antigenically Important Regions
Source: PLoS One. 2011 Mar 18;6(3):e18016. doi: 10.1371/journal.pone.0018016 (PMC3060926; doi:10.1371/journal.pone.0018016)

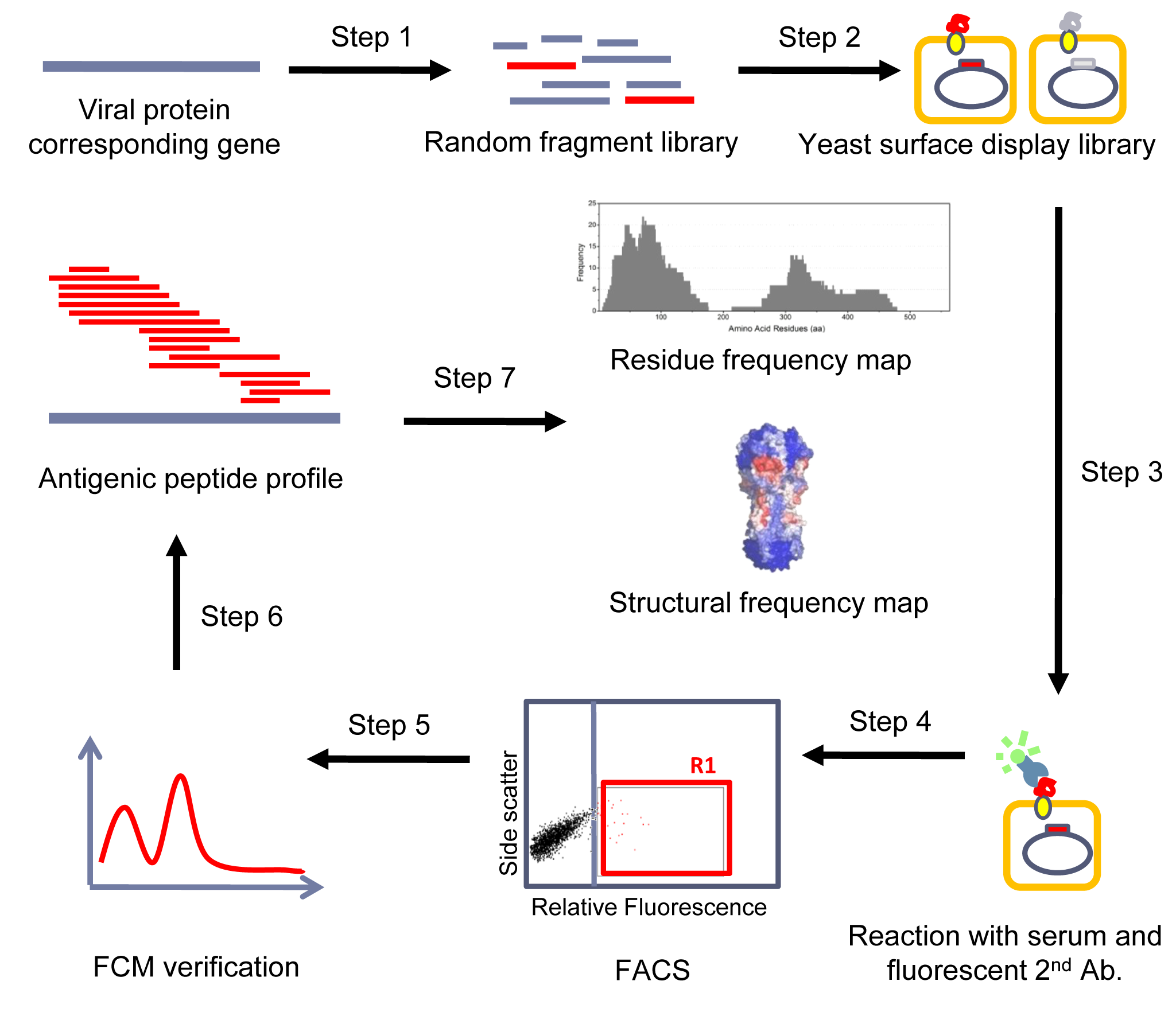

Supplement: Figure S1 — Schematic outline of the screening approach. In Step 1, the gene encoding the viral protein H1N1 HA was amplified, digested and re-assembled to generate the fragment library (the red and grey segments indicate the antigenic and non-antigenic peptides, respectively). In Step 2, the fragment library was ligated into the display vector pCTCON-T, transformed into yeast cells and induced for expression. In Step 3, the yeast cells expressing random peptides were incubated with antisera and fluorescence-labeled second antibodies and subjected to FACS in Step 4. Afterward in Step 5, the gene fragments were isolated from the sorted cells and sequenced. The in-frame sequences were re-transformed into yeast cells and verified by FCM detection individually. In Step 6, an antigenic peptide profile was extracted for the sequences corresponding to the antigenic peptides, based on which the residue frequency and structural frequency maps were derived in Step 7. (TIF) [file pone.0018016.s001.tif]

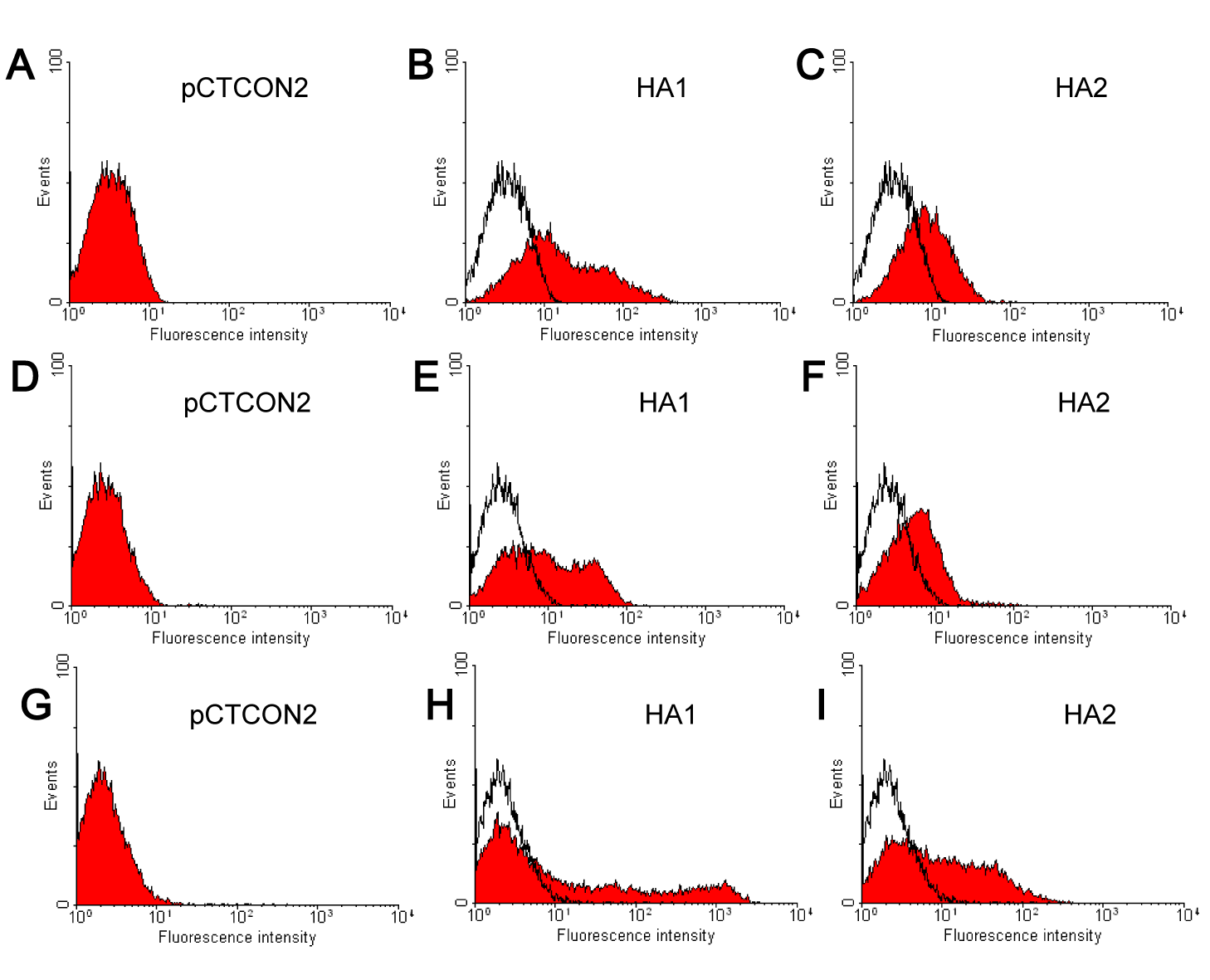

Supplement: Figure S2 — FCM detection of HA1 and HA2 displayed on yeast. Panels A, D, and G show FCM histograms of yeast cells expressing negative control vector pCTCON-2 stained with mouse antisera (and labeled by anti-Mouse IgG FITC), goat antisera (labeled by anti-Goat IgG FITC), and human plasma samples (labeled by anti-Human IgG PE), respectively. HA1 positive controls are similarly shown in panels B, E and H, and HA2 positive controls in panels C, F and I. (TIF) [file pone.0018016.s002.tif]

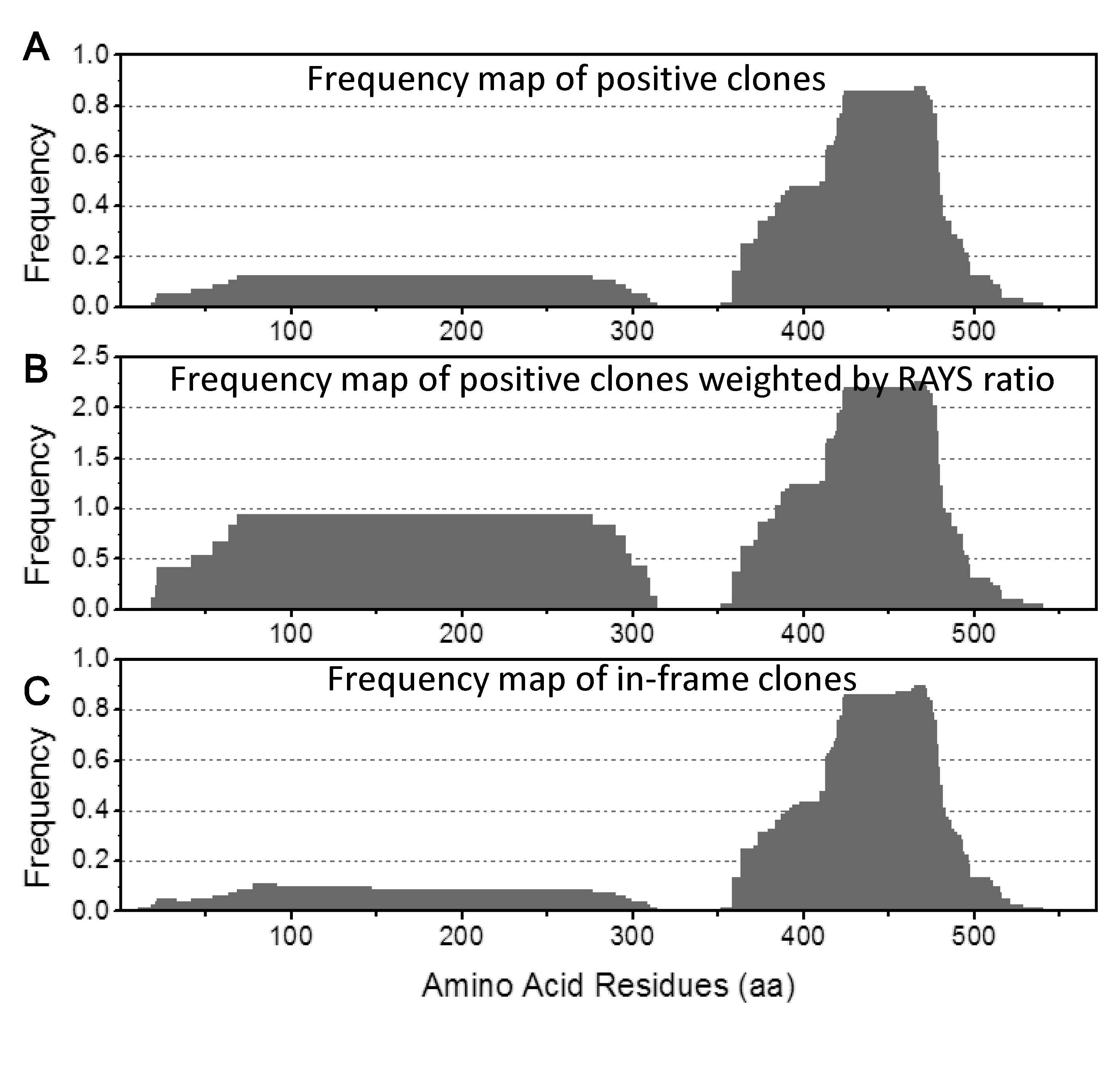

Supplement: Figure S3 — Statistical analyses of peptides from screening against mouse antisera. (A) Frequency map for each residue appearing in the 56 positive antigenic peptides (RAYS ratio ≥2). This figure is same as Fig. 4, panel A. (B) Frequency map for each residue appearing in the 56 positive antigenic peptides, but weighted by the respective RAYS ratio. (C) Frequency map for each residue appearing in all 82 in-frame peptides sorted from the library before they were individually verified by FCM. (TIF) [file pone.0018016.s003.tif]

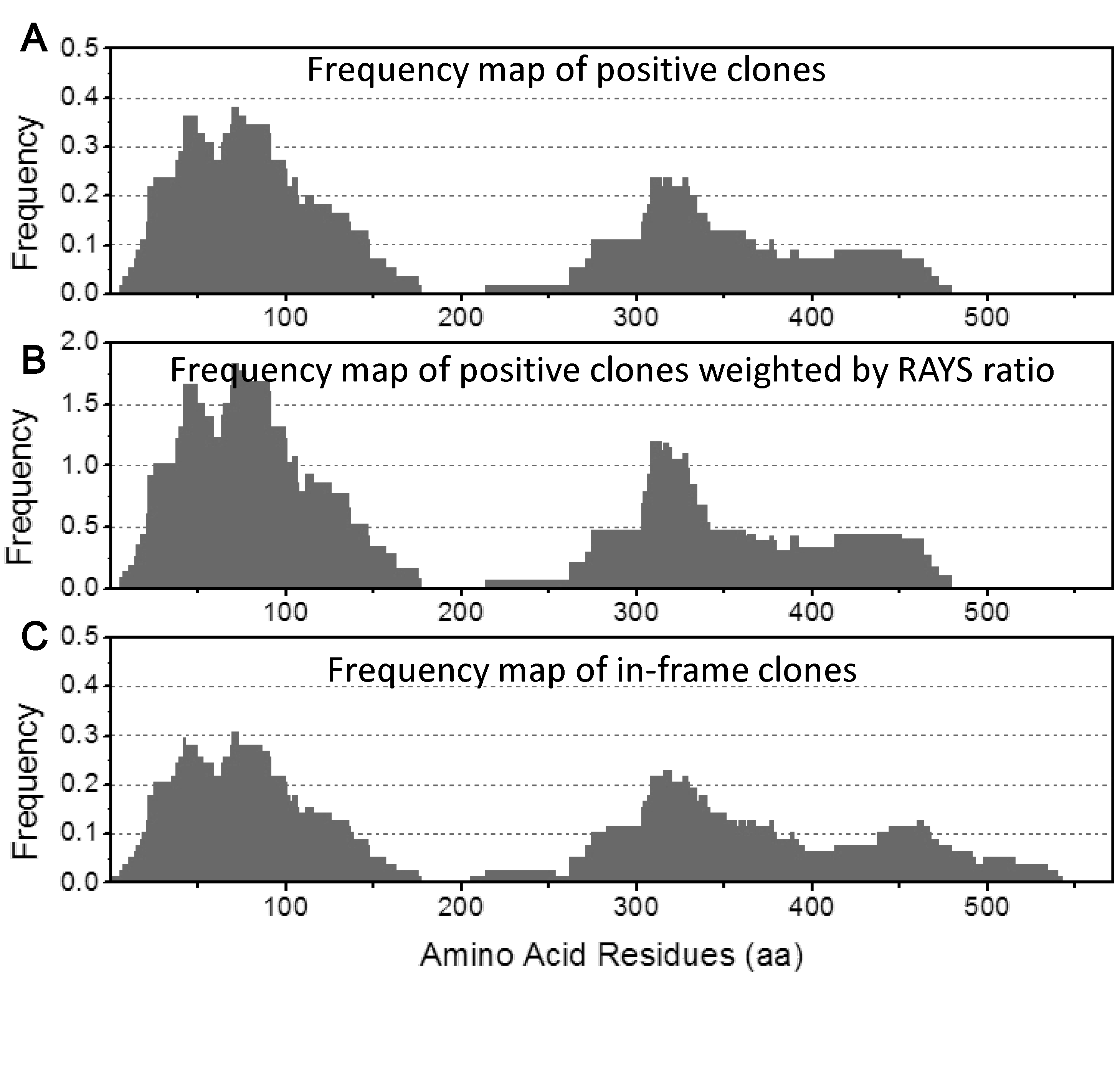

Supplement: Figure S4 — Statistical analyses of peptides from screening against goat antisera. (A) Frequency map for each residue appearing in the 55 positive antigenic peptides (RAYS ratio ≥2). The figure is same as Fig. 4, panel C. (B) Frequency map for each residue appearing in the 55 positive antigenic peptides, but weighted by the respective RAYS ratio. (C) Frequency map for each residue appearing in all 78 in-frame peptides sorted from the library before they were individually verified by FCM. (TIF) [file pone.0018016.s004.tif]

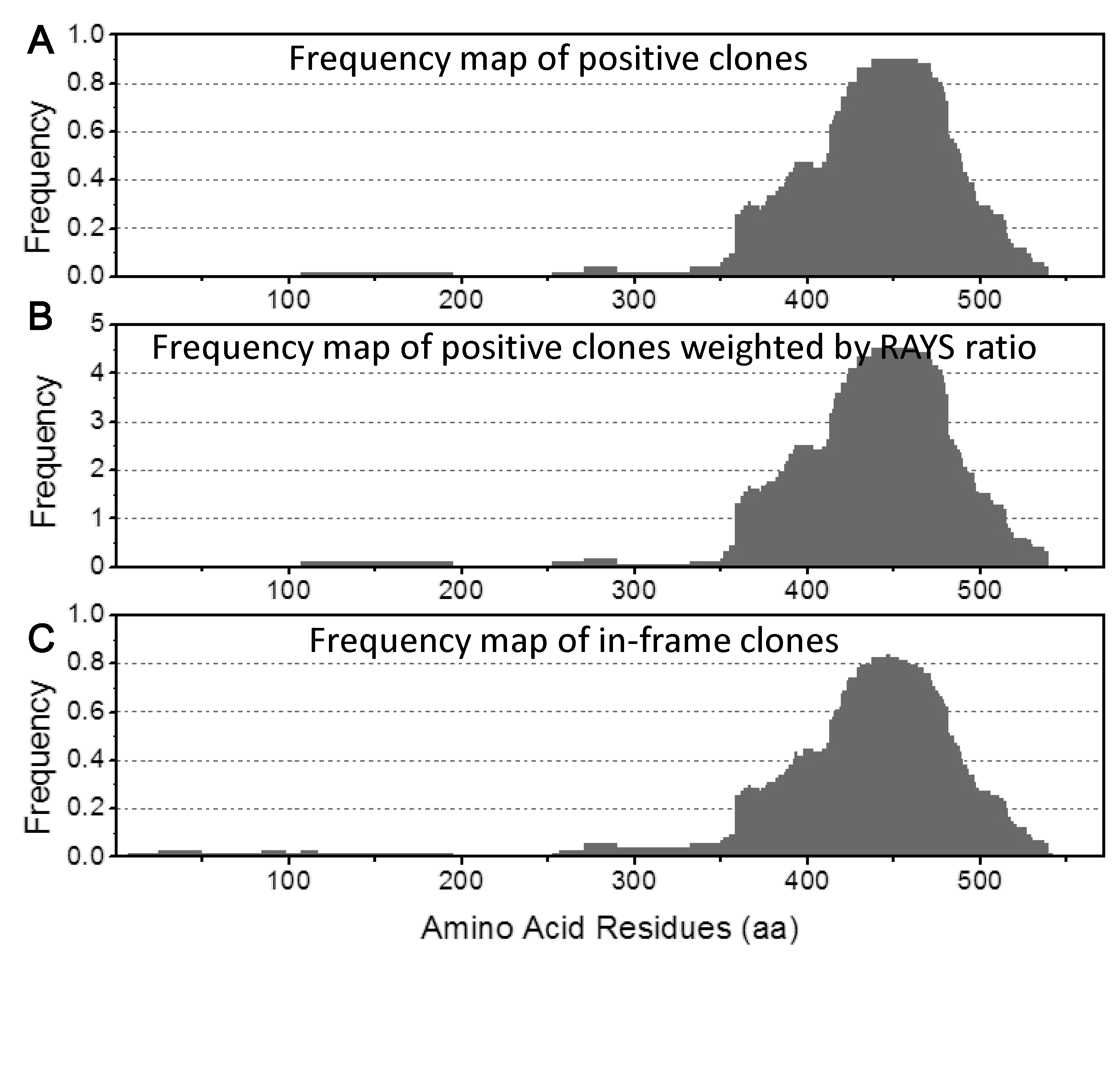

Supplement: Figure S5 — Statistical analyses of peptides from screening against human plasma. (A) Frequency map for each residue appearing in the 51 positive antigenic peptides (RAYS ratio ≥2). The figure is same as Fig. 4, panel E. (B) Frequency map for each residue appearing in the 51 positive antigenic peptides, but weighted by the respective RAYS ratio. (C) Frequency map for each residue appearing in all 74 in-frame peptides sorted from the library before they were individually verified by FCM. (TIF) [file pone.0018016.s005.tif]

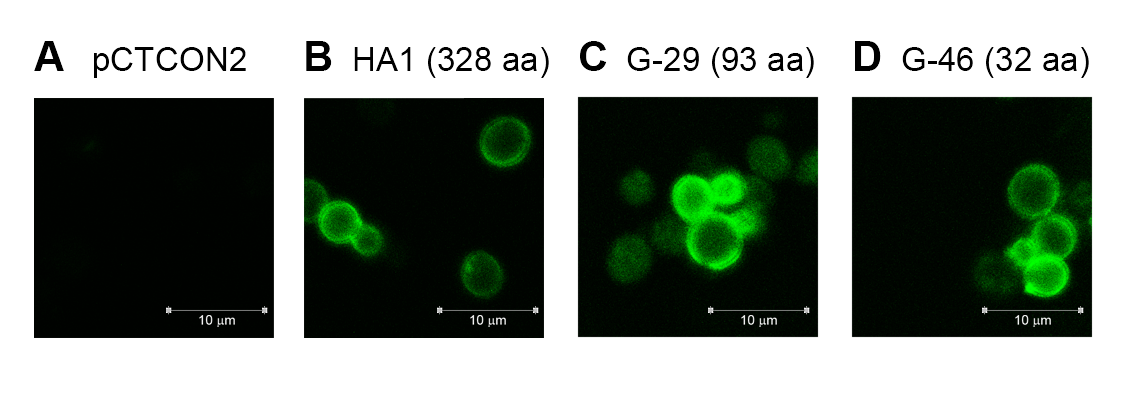

Supplement: Figure S6 — Fluorescence confocal microscopic images of yeast cells displaying antigenic peptides. Binding of the antibodies in the goat antisera to the yeast cells displaying the control vector pCTCON-2 (A), HA1 (B), and antigenic peptides G-29 (C), G-46 (D) (see also Fig. 3, panel B) were visualized by using a FITC-labeled anti-goat IgG secondary antibody. The lengths of the antigenic peptides are shown in brackets. (TIF) [file pone.0018016.s006.tif]

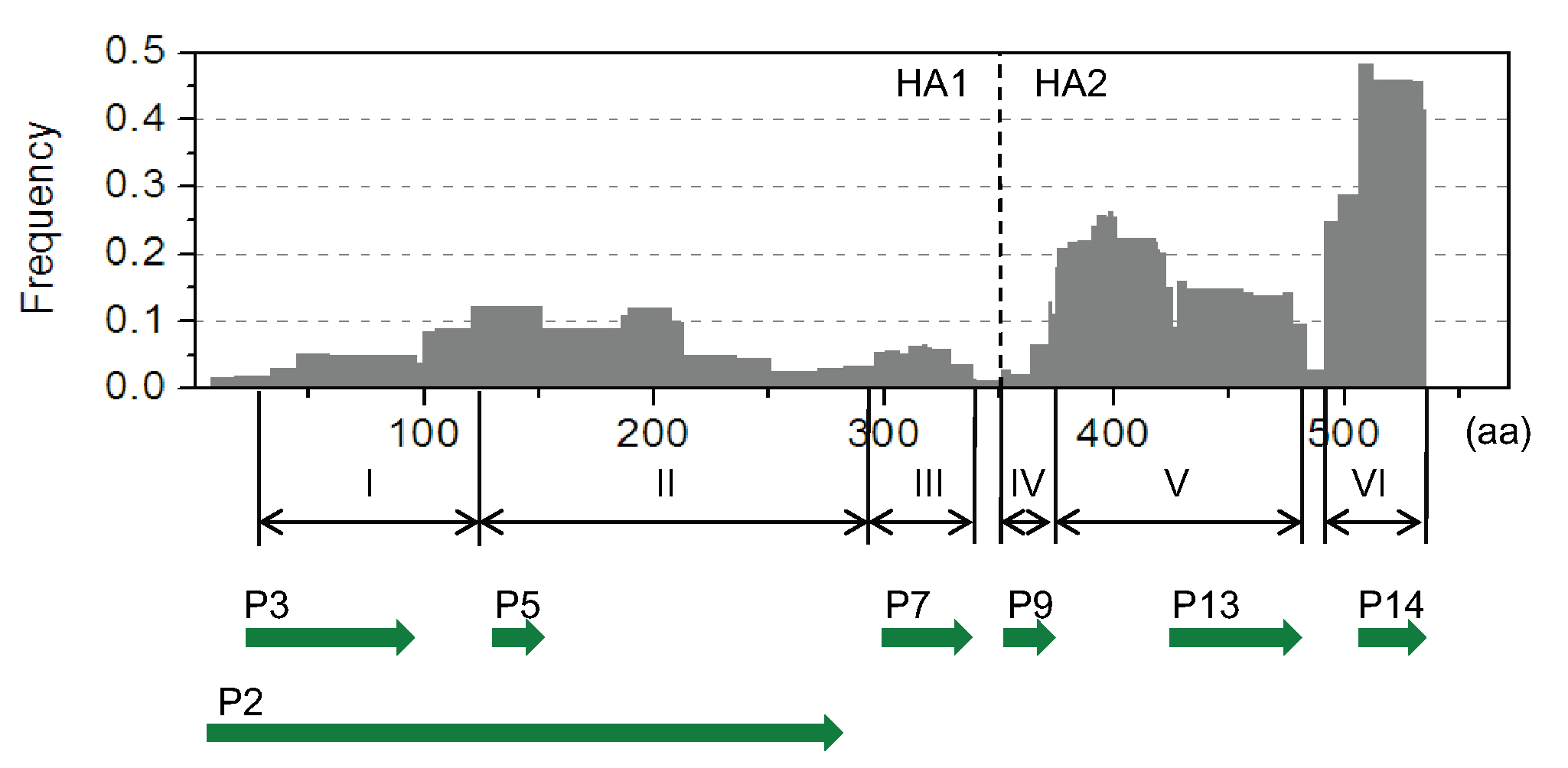

Supplement: Figure S7 — Statistical analyses of antigenic peptides based on the phage panning results shown by Khurana et al. [15] . The x-axis represents all H5N1 HA amino acid residues. The y-axis shows the normalized frequency of individual residue appearing in the 784 antigenic peptides (39 unique sequences) obtained from panning against H5N1 avian influenza convalescent sera. The six clusters (I–VI) defined by Khurana et al. are graphically represented below the x-axis. Several representative antigenic peptides are also shown as green arrows (numbered according to Khurana et al.). Even though the antigenic peptides were enriched multiple times during the screening process and thus these peptides are less diverse and might be biased in sequences, several peaks are clearly identifiable, and predominantly in the HA2 region. (TIF) [file pone.0018016.s007.tif]
