# Supplementary material for: Screening of Random Peptide Library of Hemagglutinin from Pandemic 2009 A(H1N1) Influenza Virus Reveals Unexpected Antigenically Important Regions
Source: PLoS One. 2011 Mar 18;6(3):e18016. doi: 10.1371/journal.pone.0018016 (PMC3060926; doi:10.1371/journal.pone.0018016)
Supplement: Table S1 — H1N1 virus neutralization assay. (DOC) [file pone.0018016.s008.doc]

**Table S1. H1N1 virus neutralization assay***.

| Serum/plasma sample | Absorbent | Neutralization titer IC_50_ |
| --- | --- | --- |
| Goat anti-virus sera | pCTCON-2 | 1:200 |
|  | HA1 | <1:50 |
|  | HA2 | <1:50 |
|  | G-15 (residues 38-136) | <1:50 |
|  | G-55 (residues 413-479) | <1:50 |
| Human anti-vaccine plasma | pCTCON-2 | 1:400 |
|  | HA1 | 1:50 |
|  | HA2 | 1:100 |
|  | H-11(residues 359-479) | 1:100 |

* A gradual lose in the neutralization activities of the serum samples during the prolonged absorption process (48 h in total) was observed, which resulted in low titer values insufficient to quantitatively distinguish the likely differences in the neutralization titers among the short peptides and the HA1 and HA2.
